# The Importance of Right Ventricular Lead Positioning in Determining Outcomes of Cardiac Resynchronization Therapy

**DOI:** 10.19102/icrm.2017.080601

**Published:** 2017-06-15

**Authors:** Jack Xu, Joseph Wong, Thomas E. Watts, Sirisha Reddy, Asif Sewani, Hakan Paydak

**Affiliations:** ^1^College of Medicine, University of Arkansas for Medical Sciences, Little Rock, AR; ^2^Department of Internal Medicine, Rutgers Robert Wood Johnson Medical School, New Brunswick, NJ; ^3^Division of Cardiology, University of Arkansas for Medical Sciences, Little Rock, AR; ^4^Department of Internal Medicine, University of Arkansas for Medical Sciences; ^5^Division of Cardiology, University of Alabama Birmingham School of Medicine, Birmingham, AL

**Keywords:** Cardiac resynchronization therapy, right ventricular lead positioning

## Abstract

Cardiac resynchronization therapy is known to improve clinical outcomes in patients with heart failure and left ventricular dyssynchrony. However, the optimal positioning of the right ventricular lead is unknown, and there is conflicting data on the acute hemodynamic effects and long-term outcomes. Here, we present a case of a patient who underwent implantation of a dual-chamber pacemaker for complete heart block, but who after three months, still had symptoms consistent with New York Heart Association (NYHA) Class IV heart failure. After optimal medical therapy failed and a left ventricular lead was placed, he still remained symptomatic, so the right ventricular lead was repositioned from the right ventricular outflow tract to the right ventricular apex. Afterwards, the patient’s symptoms improved from NYHA Class IV to NYHA Class II, and his left ventricular ejection fraction improved from 20% to 45%.

## Introduction

Cardiac resynchronization therapy (CRT) improves clinical outcomes in patients with heart failure and left ventricular dyssynchrony.^1^ However, only two-thirds of patients who receive CRT benefit from this treatment modality, and predictors include the position of the left ventricular lead, myocardial scar burden and optimal adjustment of the pacing intervals.^2,3^ Optimal position on the right ventricular lead is unknown, and there are conflicting data on the acute hemodynamic effects and long-term outcomes.^4,5^ Here, we report a case with heart failure secondary to right ventricular outflow tract (RVOT) pacing for complete heart block, which did not improve after an upgrade to a biventricular pacemaker. We repositioned the right ventricular lead from the RVOT to the right ventricular apex, and this resulted in clinical improvement, followed by radiological evidence of improved left ventricular function.

### Case description

We present an 80-year-old African-American male with type II diabetes mellitus, hypertension, and impaired renal function who presented with symptomatic complete heart block. An echocardiogram showed normal-sized atria and ventricles, and normal left ventricular function with an ejection fraction (EF) of 60%.

**Figure 1:**
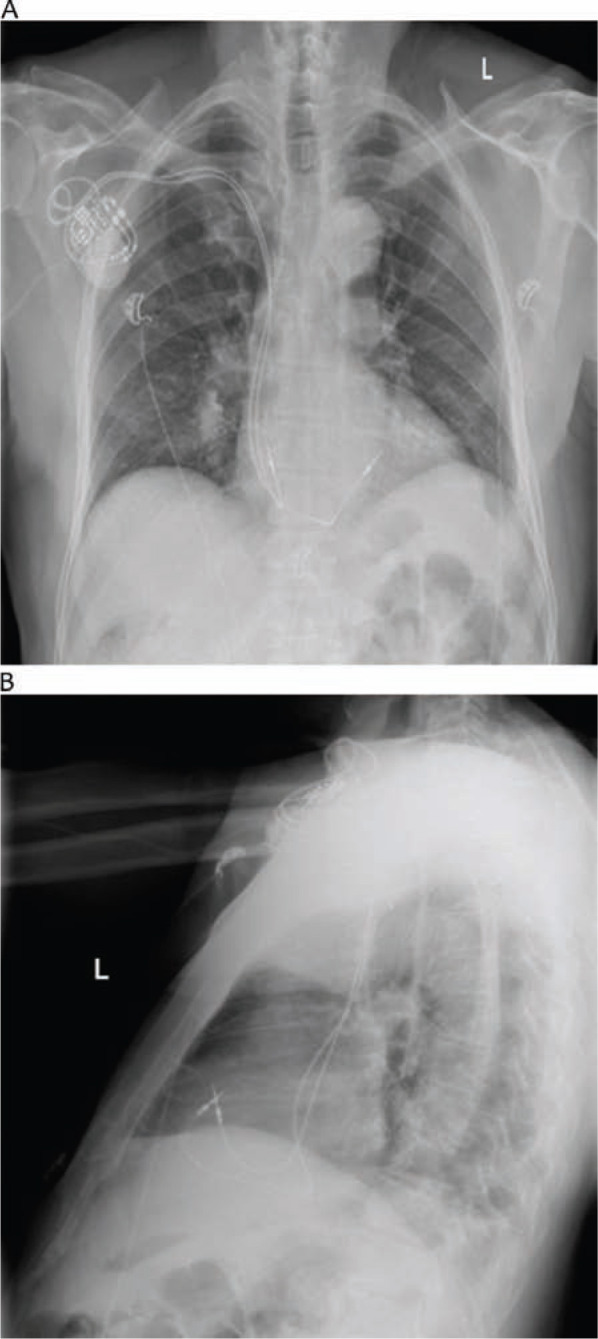
Chest radiograph of a dual-chamber pacemaker implanted with right ventricular lead in the right ventricular outflow tract. **A:** Posterioranterior view. **B:** Lateral view.

The patient underwent successful implantation of a dual-chamber pacemaker for symptomatic complete atrioven-tricular block. The right ventricular pacing lead was placed at the RVOT **(Figure 1)**. However, at a three-month follow-up visit, he demonstrated symptoms consistent with New York Heart Association (NYHA) Class IV heart failure. His brain natriuretic peptide level was 1,024 pg/ml (normal range <100 pg/ml). An echocardiogram recorded at that time showed an EF of 20% with global hypokinesis, moderate mitral regurgitation, and moderate pulmonary hypertension. Coronary angiography showed non-obstructive disease. The patient was started on guideline-directed medical therapy and titrated to optimal doses. He underwent Doppler echocardiography-guided atrioventricular (AV) and ventriculoventricular (VV) optimization, with no observed clinical benefits.

**Figure 2:**
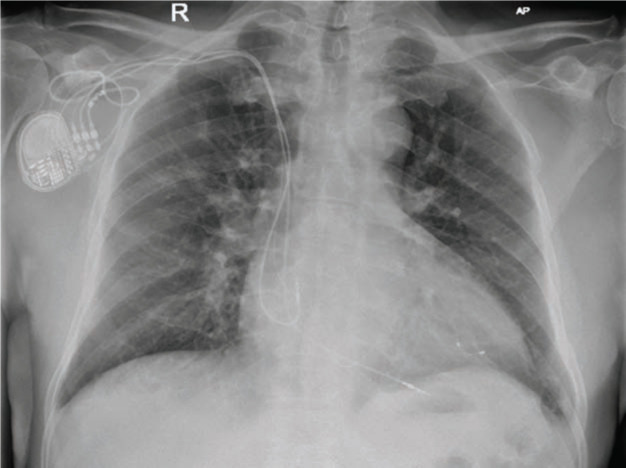
Chest radiograph of the repositioning of the right ventricular pacing lead into the right ventricular apex.

At this point, the possibility of heart failure induced by right ventricular pacing was considered, and a left ventricular lead was placed in the posteriorlateral branch of the coronary sinus three months after initial implantation of the pacemaker. Despite an upgrade to a biventricular pacemaker for resynchronization therapy, however, the patient remained symptomatic, and his left ventricular function failed to improve. It was decided then to reposition the right ventricular lead from the RVOT to the right ventricular apex. This was successfully performed seven months after the initial implantation of the pacemaker, with the hypothesis that this would alter the activation vector to promote myocardial remodeling **(Figure 2)**. One week after the right ventricular lead revision, the patient reported an improvement in symptoms. Following Doppler echocardiography-guided AV and VV optimization at the two-month follow-up, the patient’s symptoms were observed to have improved from NYHA Class IV to NYHA Class II. A subsequent multigated acquisition scan nine months after repositioning showed a left ventricular EF of 45%.

## Discussion

Even though CRT has been well studied, there is a lack of data regarding optimal right ventricular lead placement. One study has shown that RVOT pacing improves cardiac output compared with apical lead placement.^6^ Another study demonstrated that RVOT pacing provides a significant hemodynamic benefit during single-chamber pacing, when compared with apex pacing.^7^ However, this current case is an interesting one that demonstrates a lack of response to resynchronization therapy, despite the use of an ideal left ventricular lead position. In our case, the position of the right ventricular lead was the determining factor for the clinical outcome. This is in contrast to published findings from a study that randomized 32 patients with heart failure and NYHA Classes III to IV to optimized medical therapy and atrial fibrillation to RVOT or right ventricular apex pacing at one month.^8^ The pacing site was switched three months later. About 67% of them responded to cardiac resynchronization therapy, but there was no significant difference between switching of the pacing sites. In our case, the patient did not have atrial fibrillation; however, he did have a remarkable clinical improvement with right ventricular lead repositioning, confirmed by radiological evidence of improved left ventricular EF. One possible mechanism to explain the improvement in left ventricular function with the change in pacing site is a response to the change in the activation vector. Failure to respond to CRT after four months can be seen with late responders who do not end up responding after 12 months or more, which may be due to continuous reverse remodeling.^9^ Further studies regarding the role of right ventricular lead positioning in non-responders to CRT may be helpful.
